# Sofosbuvir based treatment of chronic hepatitis C genotype 3 infections—A Scandinavian real-life study

**DOI:** 10.1371/journal.pone.0179764

**Published:** 2017-07-13

**Authors:** Olav Dalgard, Ola Weiland, Geir Noraberg, Lars Karlsen, Lars Heggelund, Martti Färkkilâ, Ulla Balslev, Erika Belard, Anne Øvrehus, Mette Skalshøi Kjær, Henrik Krarup, Birgit Thorup Røge, Sofie Hallager, Lone G. Madsen, Alex Lund Laursen, Martin Lagging, Nina Weis

**Affiliations:** 1 Akershus University Hospital, Lørenskog, Norway; 2 Institute of Clinical Medicine, University of Oslo, Oslo, Norway; 3 Karolinska Institutet at Karolinska University Hospital Huddinge, Stockholm, Sweden; 4 Sørlandet sykehus, Arendal, Norway; 5 Stavanger University Hospital, Stavanger, Norway; 6 Vestre Viken, HF, Drammen, Norway; 7 Helsinki University Hospital, Helsinki, Finland; 8 Copenhagen University Hospital, Herlev, Denmark; 9 Odense University Hospital, Odense, Denmark; 10 Copenhagen University Hospital, Rigshospitalet, Copenhagen, Denmark; 11 Aalborg University Hospital, Aalborg, Denmark; 12 Kolding Hospital, Kolding, Denmark; 13 Copenhagen University Hospital, Hvidovre, Denmark; 14 Køge Hospital, Køge, Denmark; 15 Department of Infectious Diseases, Aarhus University Hospital, Aarhus, Denmark; 16 Sahlgrenska Academy, University of Gothenburg, Gothenborg, Sweden; 17 Department of Clinical Medicine, Faculty of Health and Medical Sciences, University of Copenhagen, Copenhagen, Denmark; Kaohsiung Medical University Chung Ho Memorial Hospital, TAIWAN

## Abstract

**Background and aims:**

Chronic hepatitis C virus (HCV) genotype 3 infection with advanced liver disease has emerged as the most challenging to treat. We retrospectively assessed the treatment outcome of sofosbuvir (SOF) based regimes for treatment of HCV genotype 3 infections in a real life setting in Scandinavia.

**Methods:**

Consecutive patients with chronic HCV genotype 3 infection were enrolled at 16 treatment centers in Denmark, Sweden, Norway and Finland. Patients who had received a SOF containing regimen were included. The fibrosis stage was evaluated by liver biopsy or transient liver elastography. The following treatments were given according availability and local guidelines: 1) SOF + ribavirin (RBV) for 24 weeks, 2) SOF + daclatasvir (DCV) +/-RBV for 12–24 weeks, 3) SOF + pegylated interferon alpha (peg-IFN-α) + RBV for 12 weeks or 4) SOF/ledipasvir (LDV) + RBV for 12–16 weeks. The primary endpoint was sustained virological response (SVR) assessed at week 12 (SVR12) after end of treatment.

**Results:**

We included 316 patients with a mean age of 55 years (range 24–79), 70% men, 49% treatment experienced, 58% with compensated cirrhosis and 12% with decompensated cirrhosis.In the modified intention to treat (mITT) population SVR12 was achieved in 284/311 (91%) patients. Among 26 treatment failures, five had non-response, 3 breakthrough and 18 relapse. Five patients were not included in the mITT population. Three patients died from reasons unrelated to treatment and two were lost to follow-up. The SVR12 rate was similar for all treatment regimens, but lower in men (p = 0.042), and in patients with decompensated liver disease (p = 0.004).

**Conclusion:**

We found that sofosbuvir based treatment in a real-life setting could offer SVR rates exceeding 90% in patients with HCV genotype 3 infection and advanced liver disease.

## Introduction

Approximately 100,000 individuals are infected with hepatitis C virus (HCV) in Scandinavia (Denmark, Finland, Norway, Sweden) of whom 50% have genotype 3 [1, 2]. Patients with HCV genotype 3 seem to have a more rapid progression to cirrhosis and its complications than patients infected with other genotypes [3].

Since 2011 new direct acting antivirals (DAAs) against HCV have been available [4]. The first generation protease inhibitors lacked effect against genotype 3 whereas the nucleotide analogue NS5B polymerase inhibitor sofosbuvir (SOF) had pan-genotypic activity including effect on genotype 3 [5] [6]. SOF given during 24 weeks in combination with ribavirin (RBV) for genotype 3 infections offered sustained virologic response (SVR) 12 weeks after end of treatment (SVR12) in 90% of non-cirrhotic patients but in only 60% in cirrhotic patients [7].

In vitro studies with the NS5A inhibitor daclatasvir (DCV) also indicated high efficacy against all genotypes including genotype 3 [8]. In the ALLY-3 study SOF+DCV without RBV was given for 12 weeks and yielded SVR12 in 94% in non-cirrhotic genotype 3 patients but only in 63% of those with cirrhosis [9]. In the ALLY-3+ study, SOF+DCV+RBV was given for 12 or 16 weeks, to treatment naïve and experienced patients with stage F3-F4 fibrosis, and SVR was achieved in 31/36 (86%) of F4 patients with no improvement with treatment prolonged to 16 weeks [10].

A second NS5A inhibitor, ledipasvir (LDV) has been combined with SOF in a fixed dose combination (FDC) tablet, administered once daily. This therapy is highly effective against HCV genotype 1 infections, also in cirrhotic patients [11]. Little data have been published for genotype 3 infections, but both in vitro studies and phase 2 studies indicate that LDV is less effective than DCV in genotype 3 infections [12, 13].

The aim of this study was to document the effect of sofosbuvir based regimens in patients treated for HCV genotype 3 infection in a real life setting in Scandinavia.

## Materials and methods

This retrospective, multicenter cohort study included HCV genotype 3 infected patients ≥18 years of age old who had received at least one dose of SOF in Denmark, Norway, Finland or Sweden. Organ transplanted and HIV or HBV co-infected patients were excluded. If patients had received treatment with a SOF containing regime more than once, only the first treatment was included in the present analysis. In Sweden the Regional Ethical Review Board, Stockholm, Sweden (Regionala etikprövningsnämnden i Stockholm) and Regional Ethical Review Board, Gothenburg, Sweden (Regionala etikprövningsnämnden i Göteborg) reviewed to the study and granted permission. In Denmark the Danish Data Protection Agency approved the collection of data (jnr. 1-16-02-453-12).

The institutional review boards (IRBs) at each of the participating study centers in Norway and Finland (Akershus University Hospital; Sørlandet sykehus, Arendal; Stavanger University Hospital and Vestre Viken, HF, Drammen, Norway and Helsinki University Hospital, Finland) approved the study.

### Treatment

The following treatment regimens were used according to national guidelines and/or availability of DAA drugs: 1) SOF + RBV for 24 weeks, 2) SOF + DCV +/-RBV for 12–24 weeks, 3) SOF/LDV +RBV for 12–24 weeks or 43) SOF + RBV + pegylated interferon alpha (peg-IFN-α) for 12 weeks.

### Virology

Virologic analyses were performed at the local laboratories. Plasma HCV RNA was determined using Cobas AmpliPrep/COBAS TaqMan HCV Test (Roche Diagnostics, Branchburg, NJ), which quantifies HCV RNA with a limit of detection (LOD) of 15 IU/mL or Abbott RealTime HCV with a LOD of 12 IU/mL (Abbott GmbH, Wiesbaden, Germany).

Viral genotype was determined with a hybridization technique (VERSANT HCV Genotype Assay (LiPA, Bayer HealthCare LLC, Tarrytown, NY, USA)) or by using a primer specific RT-PCR TaqMan method [14] or Sanger sequencing of the C-E1 region of HCV [15].

### Assessments

Primary efficacy endpoint was SVR, defined as undetectable HCV RNA 12 weeks after end of treatment (SVR12). Virologic breakthrough during treatment was defined as two consecutive HCV RNA results of more than 1 log_10_ IU per milliliter above the nadir HCV RNA level. Virologic relapse was defined as a detectable HCV RNA level after end of treatment in a patient who had had undetectable HCV RNA during treatment. Non-response was defined as HCV RNA being detectable at the end of treatment in a patient who did not experience virologic breakthrough.

Liver fibrosis was assessed by transient liver elastography (TE) or liver biopsy [16]. Fibrosis stage in liver biopsies was staged according to the METAVIR scale [17] and median TE level >12.5 kPa was defined as cirrhosis.

### Statistics

The analysis was conducted using the SPSS v.14.0 software package. SVR12 was evaluated in modified intention-to-treat (mITT) population that included patients who received ≥1 dose of a SOF containing regimen but did not include those without virological failure who were lost to follow-up or who died from disorders unrelated to the treatment.

Descriptive statistics were performed with the chi-square test, the paired t-test or the as appropriate

A logistic regression analysis was performed with SVR12 as dependent variable, and sex, age, treatment regime and stage of disease (compensated vs. decompensated liver disease) as independent variables. A two tailed P-value <0.05 was considered significant.

## Results

In total 316 patients with HCV genotype 3 infection were treated; 129 in Denmark, five in Finland, 74 in Norway and 108 in Sweden. Patient characteristics according to treatment regimen are presented in Table 1.

**Table 1:** Characteristics of patients with HCV genotype 3 infection who received a sofosbuvir containing treatment regime (n = 316).

	All (N = 316)	SOF+RBV 24 w (N = 33)	SOF+pegIFN+RBV 12 w (N = 56)	SOF/LDV+RBV 12–24 w (N = 32)	SOF+DCV +/-RBV 12–24 w (N = 195)
**Mean age–yr**	55	55	52	51	56
**Male sex–no. (%)**	220(70.1)	23 (69.7)	40 (72.7)	22 (71.0)	135 (69.2)
**Treatment experienced–no. (%)**	154 (49.7)	20 (62.5)	23 (41.1)	11 (34.4)	101 (51.8)
**Cirrhosis–no. (%)**	212 (68.2)	23 (69.7)	29(74.2)	22 (73.3)	139 (73.2)
Child Pugh–no (%)*					
**A**	148 (45.9%)	11 (33.3)	26 (46.4)	16 (50%)	95 (48.7)
**B**	29 (9.2)	5 (15.2)	3 (5.4)	3 (9.4)	18 (9.2)
**C**	11 (3.4)	6 (18.2)	0	1 (3.1)	4 (2.1)

*Child Pugh score was available for 188 out of 212 with cirrhosis.

In the mITT population SVR12 was achieved in 285/311 (92%) patients (in 4 patients included in the mITT population follow-up data was only available at 4 week after treatment stop). Among 26 treatment failures, five had non-response, three breakthrough and 18 relapse. Five patients were not included in the mITT population, whereof two were lost to follow-up three died from reasons unrelated to treatment (one died from metastatic colon cancer, one died from liver failure and one died from a peritonitis that was complication to coloscopy).

### Predictors of SVR

**Fig 1:**
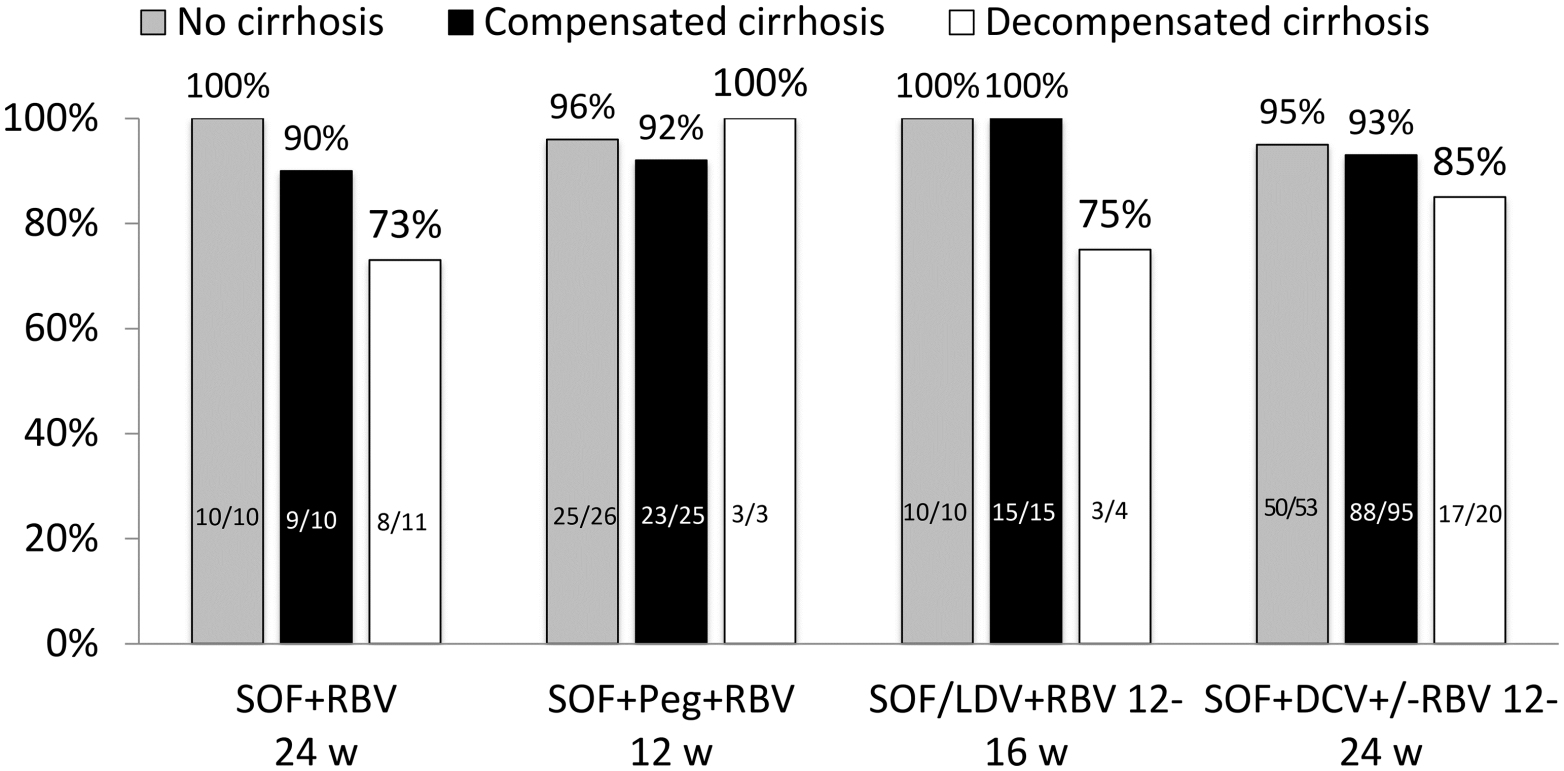
Sustained virological response (SVR) 12 weeks after end of treatment, according to different sofosbuvir based treatment regimens and stage of liver disease in patients with chronic HCV genotype 3 infection (n = 311). *Data on 24 patients with cirrhotic patients is not included due to missing CP score.

**Fig 2:**
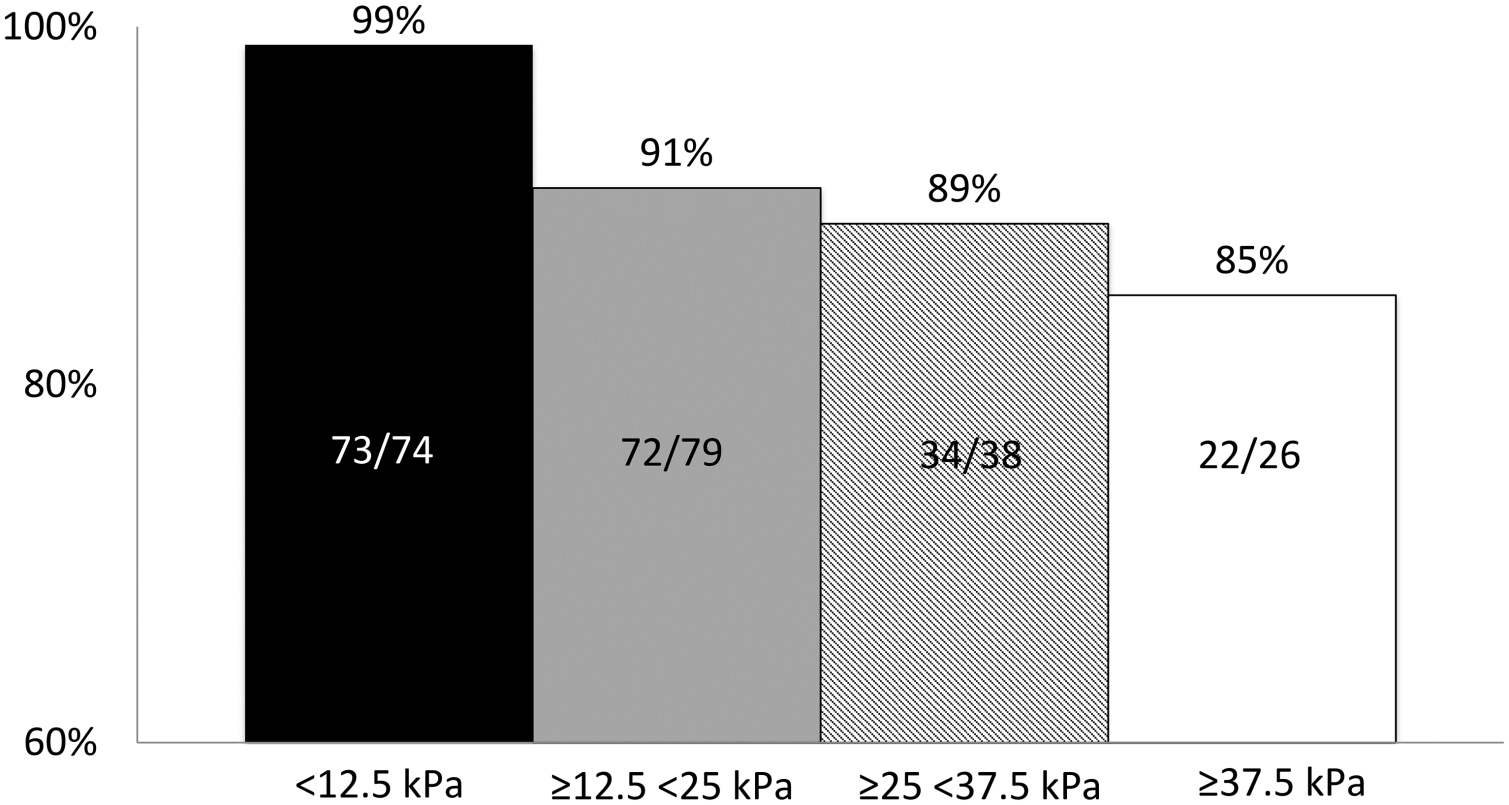
Sustained virological response (SVR) according to liver elasticity levels in patients with chronic HCV genotype 3 infection, treated with a sofosbuvir based.

The SVR12 rates were similar across all treatment regimens (Fig 1). However, the SVR12 rate was lower in men than in women (194/217 (89%) and 90/93 (97%) respectively; p = 0.042). Furthermore, a trend towards a poorer response rate was noted in patients with cirrhosis as compared to non-cirrhotic patients (190/212 (90%) and 95/99 (96%) respectively; p = 0.060). Patients with decompensated cirrhosis (Child-Pugh B or C) had lower SVR12 rates than patients with compensated liver disease (31/38 (82%) and 238/252 (94%) respectively; (p = 0.004)) (Fig 1). Similarly, increasing liver stiffness was associated with a decreased chance of achieving SVR12 (p = 0.030) (Fig 2).

In a multivariate, stepwise logistic-regression analysis, which did not include liver stiffness results, SVR12 in the mITT group was associated with female gender (p = 0.032) and the absence of decompensated liver disease (p = 0.04).

### Treatment with SOF + DCV

SOF + DCV had been administered to 193/316 (61%) patients among whom 123 (64%) also received RBV. Treatment duration was short (12–16 weeks) in 119/193 (62%) (12 weeks in 71 patients) and long (17–26 weeks) in 74/193 (38%) patients (24 weeks in 66 patients).

In the mITT group, SVR12 was obtained in 177/193 (92%) of those treated with SOF + DCV +/-RBV. SVR12 rates did not differ between those who received shorter versus longer treatment duration (111/119 (93%) and 66/74 (89%) respectively. Nor did it differ between those who did and did not receive RBV (115/123 (93.5%) versus 62/70 (88.6%), respectively). However, among those who received short treatment there was a trend towards higher SVR12 rates in patients who received RBV compared with those who did not (78/81 (96%) and 33/38 (87%) respectively; p = 0.055). In further bivariate analyses, SVR12 was independent of age, gender, treatment experience, the presence of cirrhosis, the presence of decompensated liver disease and liver elasticity.

SOF+DCV was administered to 20 patients with Child Pugh B or C and SVR was achieved in 17 (85%) of these. Treatment duration was 24 weeks in 15 (75%) and 12–16 weeks in 5 (25%) patients. RBV was administered to 12 (60%) patients.

## Discussion

In this real life Scandinavian SOF based HCV treatment study of patients with HCV genotype 3 infection, many with advanced liver disease, SVR12 was achieved in a 92%. Hence, 96% of non-cirrhotic patients achieved SVR12, which is in line with earlier studies reporting SVR12 rates between 87% and 97%, [7, 9, 18]. Furthermore, 89% of patients with cirrhosis achieved SVR12, an impressive cure rate since many of the cirrhotics had decompensation. The SVR12 rate in cirrhotics is higher than that reported in phase 2 and 3 clinical trials [9, 10].

Although adherence was not systematically monitored, we speculate that the high SVR12 rate achieved in our study was achieved by high a very high adherence rate generated by close monitoring of experienced nurses.

Only few studies have reported the effect of SOF based treatment in HCV genotype 3 infected patients with decompensated liver disease in a real life setting[19, 20]. In line with our data a German real-life study showed that SVR12 was achieved in 83% of 41 patients with genotype 3 and decompensated liver disease treated with SOF + DCV +/-RBV [19]. While the response is better than what was achived in the NHS England Expanded Access Program (EAP) that reported SVR in 68% of 192 decompensated patients with HCV genotype 3, treated with either SOF/LDV or SOF + DCV [21]. Recently, Velpatasvir (VEL), a 2^nd^ generation NS5A inhibitor with pangenotypic efficacy, has been approved in Europe and North America in a FDC tablet with SOF [22]. Among patients with compensated cirrhosis and HCV genotype 3 infection, 93% achieved SVR12 after 12 weeks treatment with SOF/VEL. However, among those with decompensated liver disease, only 50% achieved SVR12 after 12 or 24 weeks of treatment with SOF/VEL [23]. This SVR12 rate increased to 85% when RBV was added, but only 13 patients received this treatment.

The European Association for the Study of the Liver (EASL) does not recommend treating HCV genotype 3 infection with SOF/LDV as LDV has an in vitro activity against genotype 3 substantially lower than DCV [13]. DCV was not available for all of our patients during the initial study period, and thus 32 patients were treated with SOF/LDV + RBV for 12–16 weeks of whom 28 (94%) achieved SVR. Among the cirrhotic patients treated with SOF/LDV + RBV 91% achieved SVR. One could speculate that the high SVR rate we observed was caused by SOF+RBV and that LDV contributed only to a minor extent. This, however, is unlikely given the fact that the SVR rate after 12 weeks of SOF + RBV, administered to patients with HCV genotype 3 and cirrhosis, was only 34% in a phase 3 trial [6].

Male gender was associated with treatment failure, also when controlling for stage of liver disease. The ALLY-3 study, in which all patients received SOF + DCV for 12 weeks, reported a similar trend [9]. We speculate that this may reflect an immunological vulnerability in men which has also been noted in acute HCV infections, where men less often spontaneously resolve the infection than women [24].

Since this study is a multicenter, real life, non-randomized cohort study, the results must be interpreted with caution. Notably, no external monitoring of the data was performed. Despite these shortcomings, data obtained in a real world setting are a complement to randomized clinical trials, and provide important outcome data in a broader range of patients, including those with more advanced liver disease who are often excluded from pivotal registration trials.

## Conclusion

We found that sofosbuvir based treatment in a real-life setting could offer SVR rates exceeding 90% in patients with HCV genotype 3 infection and advanced liver disease.

## References

[1] Cornberg M, Razavi HA, Alberti A, Bernasconi E, Buti M, Cooper C, et al A systematic review of hepatitis C virus epidemiology in Europe, Canada and Israel. Liver Int. 2011;31 Suppl 2:30–60. Epub 2011/06/18. doi: 10.1111/j.1478-3231.2011.02539.x . 21651702 10.1111/j.1478-3231.2011.02539.x

[2] Bruggmann P, Berg T, Ovrehus AL, Moreno C, Brandao Mello CE, Roudot-Thoraval F, et al Historical epidemiology of hepatitis C virus (HCV) in selected countries. J Viral Hepat. 2014;21 Suppl 1:5–33. doi: 10.1111/jvh.12247 . 24713004 10.1111/jvh.12247

[3] Kanwal F, Kramer JR, Ilyas J, Duan Z, El-Serag HB. HCV genotype 3 is associated with an increased risk of cirrhosis and hepatocellular cancer in a national sample of U.S. Veterans with HCV. Hepatology. 2014;60(1):98–105. doi: 10.1002/hep.27095 ; 24615981 10.1002/hep.27095 PMC4689301

[4] Poordad F, McCone J Jr., Bacon BR, Bruno S, Manns MP, Sulkowski MS, et al Boceprevir for untreated chronic HCV genotype 1 infection. N Engl J Med. 364(13):1195–206. Epub 2011/04/01. doi: 10.1056/NEJMoa1010494 . 21449783 10.1056/NEJMoa1010494 PMC3766849

[5] Poordad F, McCone J Jr., Bacon BR, Bruno S, Manns MP, Sulkowski MS, et al Boceprevir for untreated chronic HCV genotype 1 infection. N Engl J Med. 2011;364(13):1195–206. Epub 2011/04/01. doi: 10.1056/NEJMoa1010494 . 21449783 10.1056/NEJMoa1010494 PMC3766849

[6] Lawitz E, Mangia A, Wyles D, Rodriguez-Torres M, Hassanein T, Gordon SC, et al Sofosbuvir for previously untreated chronic hepatitis C infection. N Engl J Med. 2013;368(20):1878–87. Epub 2013/04/24. doi: 10.1056/NEJMoa1214853 . 23607594 10.1056/NEJMoa1214853

[7] Zeuzem S, Dusheiko GM, Salupere R, Mangia A, Flisiak R, Hyland RH, et al Sofosbuvir and ribavirin in HCV genotypes 2 and 3. N Engl J Med. 2014;370(21):1993–2001. doi: 10.1056/NEJMoa1316145 . 24795201 10.1056/NEJMoa1316145

[8] Wang C, Valera L, Jia L, Kirk MJ, Gao M, Fridell RA. In vitro activity of daclatasvir on hepatitis C virus genotype 3 NS5A. Antimicrobial agents and chemotherapy. 2013;57(1):611–3. doi: 10.1128/AAC.01874-12 ; 23089758 10.1128/AAC.01874-12 PMC3535966

[9] Nelson DR, Cooper JN, Lalezari JP, Lawitz E, Pockros PJ, Gitlin N, et al All-oral 12-week treatment with daclatasvir plus sofosbuvir in patients with hepatitis C virus genotype 3 infection: ALLY-3 phase III study. Hepatology. 2015;61(4):1127–35. doi: 10.1002/hep.27726 ; 25614962 10.1002/hep.27726 PMC4409820

[10] Leroy V, Angus P, Bronowicki JP, Dore GJ, Hezode C, Pianko S, et al Daclatasvir, sofosbuvir, and ribavirin for hepatitis C virus genotype 3 and advanced liver disease: A randomized phase III study (ALLY-3+). Hepatology. 2016;63(5):1430–41. doi: 10.1002/hep.28473 ; 26822022 10.1002/hep.28473 PMC5069621

[11] Afdhal N, Zeuzem S, Kwo P, Chojkier M, Gitlin N, Puoti M, et al Ledipasvir and sofosbuvir for untreated HCV genotype 1 infection. N Engl J Med. 2014;370(20):1889–98. doi: 10.1056/NEJMoa1402454 . 24725239 10.1056/NEJMoa1402454

[12] Gane EJ, Hyland RH, An D, Svarovskaia E, Pang PS, Brainard D, et al Efficacy of Ledipasvir and Sofosbuvir, With or Without Ribavirin, for 12 Weeks in Patients With HCV Genotype 3 or 6 Infection. Gastroenterology. 2015;149(6):1454–61 e1. doi: 10.1053/j.gastro.2015.07.063 . 26261007 10.1053/j.gastro.2015.07.063

[13] Gao M. Antiviral activity and resistance of HCV NS5A replication complex inhibitors. Curr Opin Virol. 2013;3(5):514–20. doi: 10.1016/j.coviro.2013.06.014 . 23896281 10.1016/j.coviro.2013.06.014

[14] Lindh M, Hannoun C. Genotyping of hepatitis C virus by Taqman real-time PCR. J Clin Virol. 2005;34(2):108–14. doi: 10.1016/j.jcv.2005.02.002 . 16157261 10.1016/j.jcv.2005.02.002

[15] Corbet S, Bukh J, Heinsen A, Fomsgaard A. Hepatitis C virus subtyping by a core-envelope 1-based reverse transcriptase PCR assay with sequencing and its use in determining subtype distribution among Danish patients. J Clin Microbiol. 2003;41(3):1091–100. doi: 10.1128/JCM.41.3.1091-1100.2003 ; 12624035 10.1128/JCM.41.3.1091-1100.2003 PMC150254

[16] Castera L, Vergniol J, Foucher J, Le Bail B, Chanteloup E, Haaser M, et al Prospective comparison of transient elastography, Fibrotest, APRI, and liver biopsy for the assessment of fibrosis in chronic hepatitis C. Gastroenterology. 2005;128(2):343–50. Epub 2005/02/03. . 15685546 10.1053/j.gastro.2004.11.018

[17] Bedossa P, Poynard T. An algorithm for the grading of activity in chronic hepatitis C. Hepatology. 1996;24(2):289–93. doi: 10.1002/hep.510240201 8690394 10.1002/hep.510240201

[18] Lawitz E, Poordad F, Brainard DM, Hyland RH, An D, Dvory-Sobol H, et al Sofosbuvir with peginterferon-ribavirin for 12 weeks in previously treated patients with hepatitis C genotype 2 or 3 and cirrhosis. Hepatology. 2015;61(3):769–75. doi: 10.1002/hep.27567 ; 25322962 10.1002/hep.27567 PMC4365682

[19] Welzel TM, Petersen J, Herzer K, Ferenci P, Gschwantler M, Wedemeyer H, et al Daclatasvir plus sofosbuvir, with or without ribavirin, achieved high sustained virological response rates in patients with HCV infection and advanced liver disease in a real-world cohort. Gut. 2016 doi: 10.1136/gutjnl-2016-312444 . 27605539 10.1136/gutjnl-2016-312444 PMC5099229

[20] Young J, Weis N, Hofer H, Irving W, Weiland O, Giostra E, et al The effectiveness of daclatasvir based therapy in European patients with chronic hepatitis C and advanced liver disease. BMC Infect Dis. 2017;17(1):45 doi: 10.1186/s12879-016-2106-x ; 28061762 10.1186/s12879-016-2106-x PMC5219681

[21] Foster GR, Irving WL, Cheung MC, Walker AJ, Hudson BE, Verma S, et al Impact of direct acting antiviral therapy in patients with chronic hepatitis C and decompensated cirrhosis. J Hepatol. 2016;64(6):1224–31. doi: 10.1016/j.jhep.2016.01.029 . 26829205 10.1016/j.jhep.2016.01.029

[22] Foster GR, Afdhal N, Roberts SK, Brau N, Gane EJ, Pianko S, et al Sofosbuvir and Velpatasvir for HCV Genotype 2 and 3 Infection. N Engl J Med. 2015;373(27):2608–17. doi: 10.1056/NEJMoa1512612 . 26575258 10.1056/NEJMoa1512612

[23] Curry MP, O'Leary JG, Bzowej N, Muir AJ, Korenblat KM, Fenkel JM, et al Sofosbuvir and Velpatasvir for HCV in Patients with Decompensated Cirrhosis. N Engl J Med. 2015;373(27):2618–28. doi: 10.1056/NEJMoa1512614 . 26569658 10.1056/NEJMoa1512614

[24] Blackard JT, Shata MT, Shire NJ, Sherman KE. Acute hepatitis C virus infection: a chronic problem. Hepatology. 2008;47(1):321–31. doi: 10.1002/hep.21902 ; 18161707 10.1002/hep.21902 PMC2277496

